# Safety and efficacy of tract embolization using gelatin sponge particles in reducing pneumothorax after CT-guided percutaneous lung biopsy in patients with emphysema

**DOI:** 10.1186/s12890-024-03125-3

**Published:** 2024-07-09

**Authors:** Xiong Yang, Hong-Tao Cheng, Yue Huang, Yuan Guo, Hang Yuan, Yue-Qi Chen, Hai-Liang Li

**Affiliations:** 1 Department of Interventional Radiology, The Affiliated Cancer Hospital of Zhengzhou University & Henan Cancer Hospital, Zhengzhou, Henan 450008 China; 2 Department of Radiology, The Affiliated Cancer Hospital of Zhengzhou University & Henan Cancer Hospital, Zhengzhou, Henan 450008 China; 3 Department of Ultrasound, People’s Hospital of Zhengzhou University & Henan Provincial People’s Hospital, Zhengzhou, Henan 450008 China

**Keywords:** Lung biopsy, Emphysema, Pneumothorax, Tract embolization, Gelatin sponge particles

## Abstract

**Background:**

The incidence of pneumothorax is higher in patients with emphysema who undergo percutaneous lung biopsy. Needle embolization has been shown to reduce the incidence of pneumothorax in patients with emphysema. Existing studies have reported small sample sizes of patients with emphysema, or the degree of emphysema has not been graded. Therefore, the efficacy of biopsy embolization in the prevention of pneumothorax induced by percutaneous pulmonary biopsy in patients with emphysema remains to be determined.

**Methods:**

In this retrospective, controlled study, patients with emphysema who underwent CT-guided PTLB were divided into two groups: group A (*n* = 523), without tract embolization, and Group B (*n* = 504), with tract embolization. Clinical and imaging features were collected from electronic medical records and Picture Archiving and Communication Systems. Univariate and multivariate analyses were performed to identify risk factors for pneumothorax and chest tube placement.

**Results:**

The two groups did not differ significantly in terms of demographic characteristics and complications other than pneumothorax. The incidence of pneumothorax and chest tube placement in group B was significantly lower than in group A (20.36% vs. 46.12%, *p* < 0.001; 3.95% vs. 9.18%, *p* < 0.001, respectively). In logistic regression analyses, variables affecting the incidence of pneumothorax and chest tube placement were the length of puncture of the lung parenchyma (odds ratio [OR] = 1.18, 95% confidence interval [CI]: 1.07–1.30, *p* = 0.001; OR = 1.55, 95% CI: 1.30–1.85, *p* < 0.001, respectively), tract embolization (OR = 0.31, 95% CI: 0.24–0.41, *p* < 0.001; OR = 0.39, 95% CI: 0.22–0.69, *p* = 0.001, respectively), and grade of emphysema.

**Conclusions:**

Tract embolization with gelatin sponge particles after CT-guided PTLB significantly reduced the incidence of pneumothorax and chest tube placement in patients with emphysema. Tract embolization, length of puncture of the lung parenchyma, and grade of emphysema were independent risk factors for pneumothorax and chest tube placement.

**Trial registration:**

Retrospectively registered.

## Background

Lung cancer is the leading cause of cancer-related mortality in China [1, 2]. Percutaneous lung biopsy (PTLB) is an important method for obtaining histological samples for the pathological diagnosis of lung lesions [3]. Pneumothorax is the most common complication of computed tomography (CT)-guided percutaneous lung biopsy [4]. Approximately 15.0% of patients develop pneumothorax, and approximately 6.6% require chest tube insertion [5]. The occurrence of these complications is influenced by various factors, including lesion location and size, underlying lung condition, needle diameter, length traversed by the biopsy needle through the lung parenchyma, and operator experience [6–8]. Emphysema is markedly associated with the incidence of pneumothorax after PTLB [9], and the incidence rates of pneumothorax and chest tube placement after PTLB are substantially higher in patients with emphysema than in those without emphysema (41.1% vs. 24.3%) and (27.2% vs. 8.8%, respectively). Tract embolization has been recommended as a preventive measure for pneumothorax according to relevant guidelines [10]. Current methods to prevent PTLB-induced pneumothorax include injection of autologous blood patches [11–14], NaCl 0.9% solution [15], hydrogel plugs [16, 17], and gelatin sponge particle slurry [18–21].

As short-term embolization materials, gelatin sponge particles are non-antigenic, non-toxic, and absorbable. These particles have been used in surgery and vascular intervention for a long time. Gelatin sponge particles have been shown to effectively prevent the development of pneumothorax and chest tube placement by blocking the biopsy tract [18]. Although some studies have included patients with emphysema, the sample sizes were small, or the degree of emphysema was not graded. The efficacy of biopsy tract embolization in preventing PTLB-induced pneumothorax in patients with emphysema needs to be clarified. Therefore, in the current study, we aimed to evaluate the efficacy and safety of biopsy tract embolization using gelatin sponge particles to reduce PTLB-induced pneumothorax in patients with emphysema.

## Materials and methods

### Study population

This retrospective study was approved by our institutional review board and ethics committee (Approval number: 2,017,002). The requirement for informed consent was waived.

**Fig. 1:**
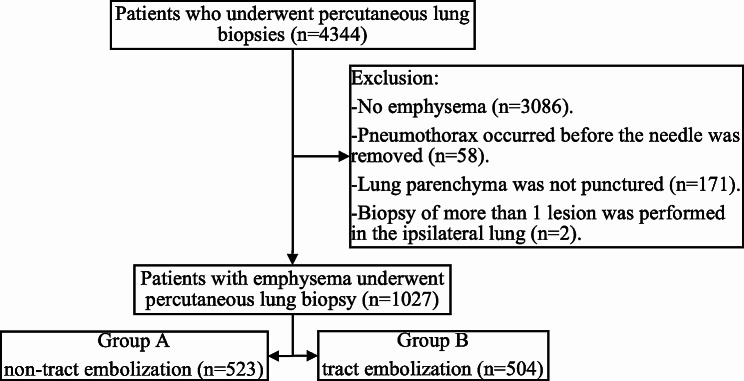
Flow Chart of the study

In April 2022, biopsy tract embolization was initiated during PTLB at our center to reduce the incidence of pneumothorax. Data of patients with emphysema who underwent PTLB without tract embolization between April 2021 and March 2022 and those with tract embolization between April 2022 and March 2023 were analyzed. Patients with emphysema who underwent PTLB were included in the study. Exclusion criteria were as follows: (1) pneumothorax that occurred before the needle was removed; (2) unpunctured lung parenchyma; and (3) biopsy of more than one lesion in the ipsilateral lung (Fig. 1). The suitability of patients to undergo transbronchial biopsy (TBB) or transbronchial needle aspiration (TBNA) was assessed by the endoscopic center before CT-guided biopsy. Of the selected patients, only 31 (3.02%) underwent TBB or TBNA; however, the results failed to establish a definitive pathological diagnosis of lung lesions.

### **Grades of emphysema**

**Fig. 2:**
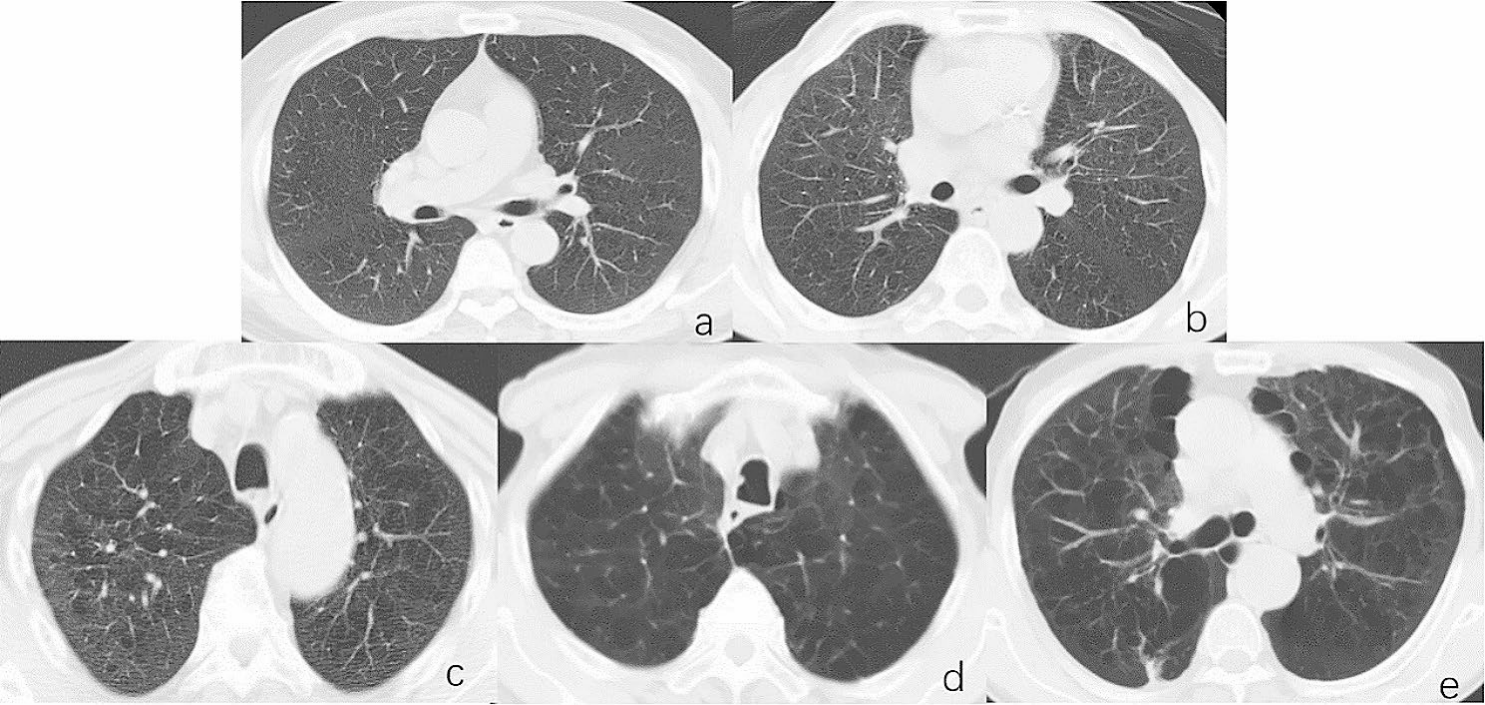
Unenhanced axial CT images showing emphysema severity grades. **(a)** Lung window of CT image showing trace centrilobular emphysema occupying < 0.5% of a lung zone. **(b)** Image shows mild centrilobular emphysema involving an estimated 0.5-5% of lung zone. **(c)**Image showing moderate centrilobular emphysema occupying more than 5% of any lung zone. **(d)** Image showing confluent centrilobular emphysema without extensive overexpansion of secondary pulmonary lobules or distortion of pulmonary architecture. **(e)** Image showing advanced destructive emphysema with overexpansion of secondary pulmonary lobules and distortion of pulmonary architecture. CT: computed tomography

The emphysema grade was evaluated using a structured visual classification system for pulmonary parenchymal emphysema proposed by the Fleischner Society [22]. This system classifies parenchymal emphysema as trace centrilobular emphysema (CLE), mild CLE, moderate CLE, confluent CLE, or advanced destructive emphysema (ADE) using a 5-point sequential grading system (Fig. 2). Two experienced chest radiologists performed a visual analysis of all patients based on the classification system of the Fleischner Association.

### Extent and management of complications

According to the Society of Interventional Radiology guidelines [23], PTLB-induced complications can be classified as minor or major. Minor complications require no therapy or nominal treatment and have no consequences. Major complications require appropriate therapy and can result in prolonged hospitalization or even permanent adverse sequelae or death.

### Percutaneous lung biopsies and tract embolization procedure

All PTLB procedures were performed by chest radiologists with more than 5 years of experience in PTLB and chest tube placement under CT guidance (Large Bore CT Machine; GE, Boston, MA, USA). A disposable coaxial biopsy needle (Max-Core™ Disposable Core Biopsy Instrument, 18G×20 cm; BD Company, Franklin Lakes, NJ, USA) was used. Written informed consent was obtained from all patients prior to the biopsy procedure. Before the procedure, an unenhanced chest CT was performed to determine the puncture point on the body surface, select the appropriate puncture path, and calculate the insertion length.

All patients breathed freely during the procedure, and their vital signs were monitored. The patients underwent a biopsy under local anesthesia. The biopsy core needle was inserted discontinuously in a “stepwise” manner following a predetermined puncture path under the supervision of CT, with appropriate adjustments. All patients were sampled 3–4 times to ensure that sufficient material was obtained for pathological diagnosis. The needle was removed directly after sampling in the non-tract-embolization group (group A). In the embolization group (group B), gelatin sponge particles (1000–1400 μm; Alicon Medical Company, Hangzhou, China) were thoroughly mixed with 5 ml of saline and inhaled into a 5 ml syringe. The gelatin sponge particle slurry was injected evenly through the coaxial biopsy sheath, and the sheath of the biopsy needle was evenly withdrawn until it was removed from the skin.

**Fig. 3:**
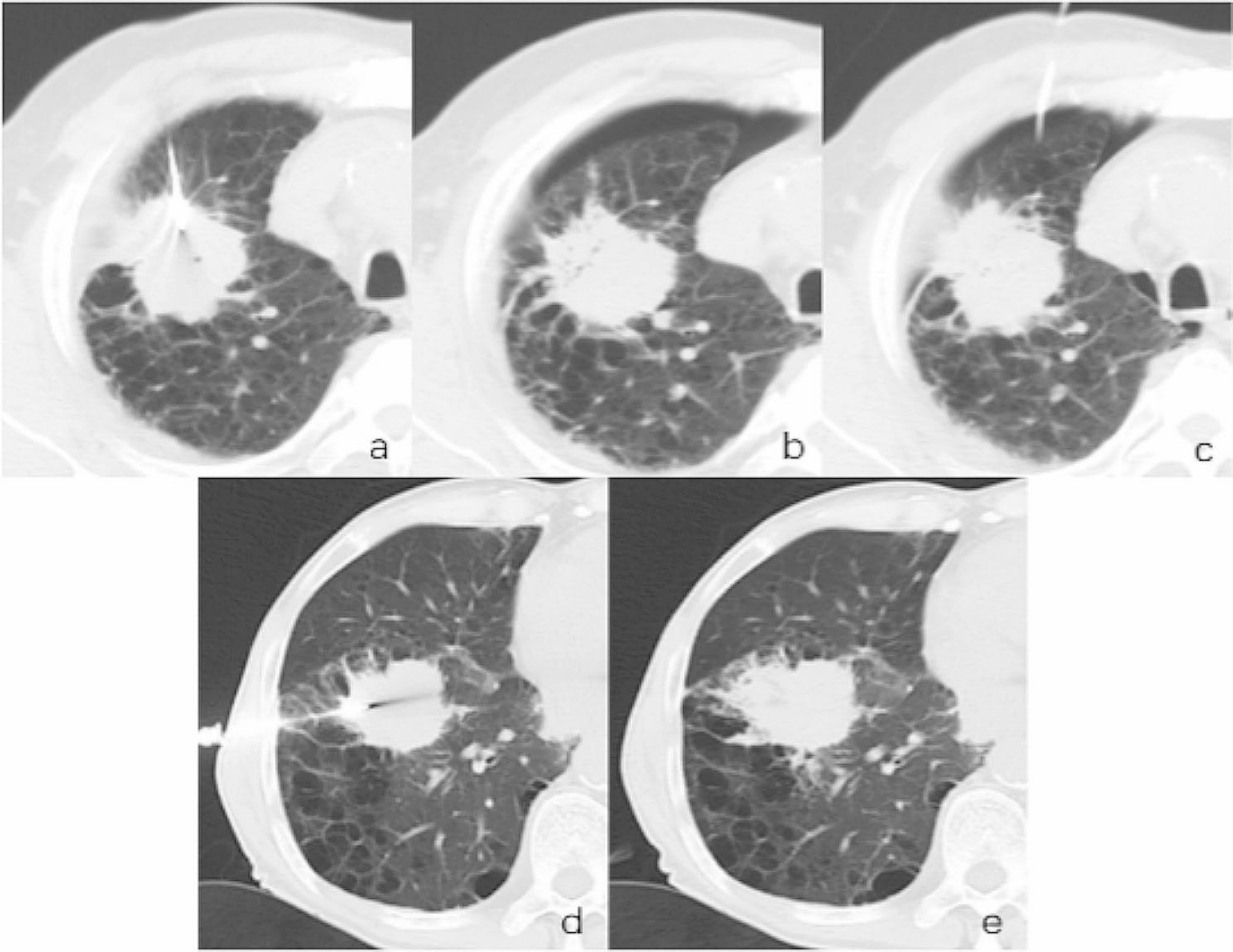
Unenhanced axial CT images **(a) -(c)** images of a patient (68Y/M) with grade e emphysema who did not undergo tract embolization during the procedure, and images **(d) - (e)** images of another patient (66Y/M) with grade e emphysema who underwent tract embolization during the procedure. **(a)** Image showing the insertion of the biopsy needle into the lung lesion. **(b)** Image showing the presence of major pneumothorax after the biopsy needle had been removed. **(c)** Image showing that the chest tube was placed and the gas inside the chest cavity was aspirated. **(d)** Image showing biopsy needle insertion into lung lesion. **(e)** Image showing that no pneumothorax occurred after the biopsy needle was removed. CT: computed tomography

Finally, an unenhanced chest CT was performed to determine the occurrence of complications. If minor or no complications occurred, no therapy or nominal treatment was administered. If major complications were detected, appropriate treatment was administered immediately (Fig. 3) [24, 25]. After the procedure, patients were maintained with biopsy side downward, ECG was closely monitored for 8 h, and nasal oxygen therapy was administered.

### Analysis of biopsy images

Two experienced chest radiologists reviewed the cases in the Picture Archiving and Communication System (PACS). They extracted radiological characteristics of the target lesion, including the lesion location, length of puncture of the lung parenchyma, emphysema grade, position during the procedure, and whether complications occurred and corresponding treatment methods. Discordances between analysts were adjudicated by another thoracic radiologist.

### Statistical analysis

Continuous variables are presented as means and standard deviations (SD), and categorical variables are expressed as numbers and frequencies. The Mann–Whitney U test was used to compare continuous variables, and the chi-squared test and Fisher’s exact test were used to compare categorical variables. Univariate and multivariate analyses were performed to assess the incidence of pneumothorax, with odds ratios (ORs) and 95% confidence intervals (CIs) reported as appropriate. Statistical significance was set at *p* < 0.05. Statistical analysis was performed using SPSS 26.0 software (IBM Corp., Armonk, NY, USA).

## Results

In total, 1027 patients were included in statistical analyses, with 523 patients in group A (non-tract embolization; male: female 370:153; age: 67.72 ± 7.73 years) and 504 patients in group B (tract embolization; male: female 359:145; age: 68.21 ± 7.40 years). Table 1 summarizes the patient demographic characteristics and the incidence of complications. There were no significant differences between the two groups in terms of age, sex, grade of emphysema, lesion location, lesion size, length of puncture of the lung parenchyma, or patient position during the procedure. In terms of complications, there were no statistically significant differences in the incidence of pulmonary hemorrhage and hemoptysis, pleural reaction, and systemic air embolism between the two groups. The incidence of pneumothorax was significantly higher in group A than in group B (46.12% vs. 20.36%, *p* < 0.001), and the incidence of chest tube placement was also significantly higher in group A than in group B (9.18% vs. 3.95%, *p* < 0.001).

**Table 1:** Clinical and imaging characteristics of the biopsy procedures

	Non-tract embolization (group A)	Tract embolization (group B)	
*N* = 1027	*N* = 523	*N* = 504	*P* value
Age (years), mean ± SD	67.72 ± 7.73	68.21 ± 7.40	0.27^*^
Sex (male/female)	370/153	359/145	086^→^
Gades of emphysema, n			0.99^§^
Trace CLE Mild CLE Moderate CLE Confluent CLE ADE	304 161 46 10 2	292 157 43 11 1	
Localization, n			0.24^→^
Right lung upper lobe Right lung mid lobe Right lung lower lobe Left lung upper lobe Left lung lower lobe	145 25 112 143 98	167 19 100 142 76	
Size (mm), mean ± SD	31.51 ± 15.00	30.10 ± 13.58	0.24^*^
Position, n Supine Prone Lateral	276 239 8	282 220 2	0.13^§^
Length of puncture of the lung parenchyma (mm), mean ± SD	24.99 ± 13.73	24.57 ± 13.90	0.53^*^
Complications, n (%) Pneumothorax Minor Chest tube placement PH and hemoptysis Pleural reaction Systemic air embolism	232(46.12) 184(35.18) 48(9.18) 44(8.41) 26(4.97) 0	103(20.36) 83(16.40) 20(3.95) 36(7.14) 29(5.75) 0	< 0.001^→^ 0.45^→^ 0.58^→^

(*, Mann-Whitney U test; →, Pearson’s chi-square test; §, Fisher’s exact test)

Abbreviations: CLE, Centrilobular Emphysema; ADE, Advanced Destructive Emphysema; PH, Pulmonary hemorrhage

Table 2 summarizes the results of subgroup analyses according to the grade of emphysema. With the aggravation of emphysema, the incidence of pneumothorax and chest tube placement increased in both groups. The incidence of pneumothorax and the rate of chest tube placement were significantly higher in group A than in group B for all grades of emphysema. However, there was no significant difference in the incidence of pneumothorax between the two groups in patients with confluent CLE & ADE grade emphysema.

**Table 2:** Comparison of different grades of emphysema in group A and group B

	Emphysema grade											
	Trace CLE			Mild CLE			Moderate CLE			Confluent CLE & ADE		
	A	B	P value	A	B	P value	A	B	P value	A	B	P value
	N = 304	N = 292		N = 161	N = 157		N = 46	N = 43		N = 12	N = 12	
Age (years), mean ± SD	67.14 ± 7.92	68.14 ± 7.48	0.11^*^	68.13 ± 7.26	68.38 ± 7.49	0.58^*^	69.22 ± 7.40	68.21 ± 7.16	0.26^*^	71.1 ± 9.1	67.7 ± 5.7	0.25^*^
Sex (male/female)	175/129	177/115	0.45^→^	142/19	130/27	0.17^→^	41/5	40/3	0.72^→^	12/0	12/0	
Localization, n			0.80^→^			0.13^→^			0.41^§^			0.67^§^
Right upper lobe	73	78		48	65		20	19		4	5	
Right mid lobe	19	16		5	3		1	0		0	0	
Right lower lobe	67	60		32	31		11	5		2	4	
Left upper lobe	77	81		50	44		11	14		5	3	
Left lower lobe	68	57		26	14		3	5		1	0	
Size (mm), mean ± SD	30.05 ± 13.08	29.29 ± 12.99	0.37^*^	32.58 ± 17.05	30.80 ± 14.78	0.24^*^	32.80 ± 18.36	33.14 ± 13.04	0.51^*^	36.0 ± 14.62	29.8 ± 12.00	0.14^*^
Position, n												
Supine	154	159		89	90		29	26		5	7	
Prone	146	132	0.31^§^	68	66	0.50^§^	17	17	0.80^→^	7	5	0.41^→^
Lateral	4	1		4	1		0	0		0	0	
Length of puncture of the lung parenchyma (mm), mean ± SD	23.69 ± 12.98	24.13 ± 13.57	0.76^*^	27.29 ± 15.32	26.70 ± 14.15	0.74^*^	24.80 ± 12.26	20.49 ± 14.34	0.067^*^	28.1 ± 11.63	25.3 ± 13.91	0.38^*^
Complications, n (%)												
Pneumothorax	110(36.18)	55(18.84)	< 0.001^→^	78(48.45)	28(17.83)	< 0.001^→^	33(71.74)	14(32.56)	< 0.001^→^	9(75.0)	6(50.0)	0.093^§^
Minor	99(32.57)	50(17.12)		64(39.75)	20(12.74)		17(36.96)	8(18.61)		3(25.0)	5(41.7)	
Chest tube placement	11(3.62)	5(1.71)		14(10.56)	8(5.10)		16(34.78)	6(13.95)		6(50.0)	1(8.3)	
PH and hemoptysis	25(8.22)	22(7.53)	0.76^→^	9(5.59)	7(4.46)	0.64^→^	7(15.22)	4(9.30)	0.40^→^	3(25)	3(25)	0.99^§^
Pleural reaction	14(4.61)	14(4.79)	0.91^→^	7(4.35)	10(6.37)	0.42^→^	4(8.70)	3(6.98)	0.99^§^	1(8.33)	2(16.67)	0.99^§^

(*, Mann-Whitney U test; →, Pearson’s chi-square test; §, Fisher’s exact test)

Abbreviations: CLE, Centrilobular Emphysema; ADE, Advanced Destructive Emphysema; PH, Pulmonary hemorrhage

In univariate and multivariate regression analyses (Table 3), tract embolization was associated with protection against pneumothorax (OR = 0.31, 95% CI: 0.23–0.41, *p* < 0.001) and chest tube placement (OR = 0.39, 95% CI: 0.22–0.69, *p* = 0.001). Length of puncture of the lung parenchyma and grade of emphysema were identified as independent risk factors for pneumothorax and chest tube placement. The incidence of pneumothorax increased with the degree of emphysema and length of puncture of the lung parenchyma.

**Table 3:** Univariate and multivariate regression analyses on risk factors for pneumothorax and chest tube placement

Variable	Pneumothorax						Chest tube placement					
	Univariate Analysis			Multivariate Analysis			Univariate Analysis			Multivariate Analysis		
	OR	95%CI	P value	OR	95%CI	P value	OR	95%CI	P value	OR	95%CI	P value
Sex (male/female)	1.38	0.99–1.91	0.05				0.80	0.37–1.73	0.57			
Age	0.99	0.98–1.01	0.55				1.00	0.97–1.04	0.82			
Lesion size	1.04	0.95–1.15	0.39				1.05	0.88–1.26	0.59			
Lesion location												
Right upper lobe	1.00						1.00					
Middle lobe	1.33	0.66–2.70	0.43				1.56	0.40–6.12	0.52			
Right lower lobe	0.80	0.53–1.21	0.29				0.81	0.37–1.79	0.60			
Left upper lobe	0.87	0.60–1.25	0.45				0.94	0.47–1.87	0.86			
Left lower lobe	0.89	0.57–1.38	0.60				1.09	0.44–2.72	0.86			
Length of puncture of the lung parenchyma	1.19	1.08–1.32	0.001	1.18	1.07–1.30	0.001	1.59	1.32–1.90	< 0.001	1.55	1.30–1.85	< 0.001
Emphysema grade												
Trace CLE	1.00			1.00			1.00			1.00		
Mild CLE	1.45	1.05-2.00	0.025	1.33	0.98–1.81	0.08	2.12	1.04–4.31	0.037	2.33	1.19–4.58	0.014
Moderate CLE	3.70	2.24–6.10	< 0.001	3.25	2.02–5.25	< 0.001	13.82	6.38–29.93	< 0.001	14.06	6.82-29.00	< 0.001
Confluent CLE & ADE	5.80	2.34–14.42	< 0.01	4.95	2.03–12.05	< 0.001	20.00	6.72–59.48	< 0.001	20.40	7.21–57.70	< 0.001
Position												
Supine	1.00						1.00					
Prone	1.27	0.93–1.74	0.13				0.94	0.51–1.71	0.83			
Lateral	1.96	0.53–7.25	0.32				5.26	0.88–31.26	0.068			
Tract embolization (Yes/No)	0.32	0.24–0.42	< 0.001	0.31	0.24–0.41	< 0.001	0.41	0.23–0.73	0.003	0.39	0.22–0.69	0.001

Abbreviations: CLE, Centrilobular Emphysema; ADE, Advanced Destructive Emphysema

## Discussion

In this retrospective analysis, 1027 patients with emphysema underwent CT-guided PTLB in our department between 2021 and 2022, among whom 523 patients did not undergo tract embolization, and 504 underwent tract embolization with gelatin sponge particles. The rates of pneumothorax and chest tube placement were significantly lower in the tract embolization group. The incidence of pneumothorax increased with the severity of the emphysema. Tract embolization could significantly reduce the incidence of pneumothorax and chest tube placement in patients with different degrees of emphysema with no additional complications.

Tract embolization has been proposed for decades, and several embolic materials have been reported. However, there is still no consensus regarding the materials recommended in international guidelines for this technique. The most common embolic materials are autologous blood patches prepared by mixing autologous venous blood with normal saline. Ensuring uniformly sized blood patches made using this method may be challenging, and success rates reported in the current literature vary [11–14]. Billich et al. [15] showed that injecting a 0.9% NaCl solution through the puncture tract could substantially reduce the incidence of pneumothorax. However, the authors did not specifically focus on patients with emphysema. In addition, the 0.9% NaCl solution may be easily absorbed and unevenly distributed in the tract under the influence of gravity. These factors may limit its suitability for tract embolization. Grage et al. [16] used hydrogel plugs for biopsy tract embolization, positioning 2 cm hydrogel plugs in the lung and 0.5 cm outside of the pleura, which may make it difficult to ensure consistent placement. In addition, the hydrogel plug is difficult to absorb and relatively expensive. Although it reduced the incidence of chest tube placement, the hydrogel plug did not substantially impact the incidence of pneumothorax. These results were inconsistent with those reported by Zaetta et al. [17]. Therefore, the role of hydrogel plugs in tract embolization of PTLB is yet to be confirmed.

In contrast, the injection of the sponge gelatin particle slurry was relatively simple. CT-guided PTLB followed by gelatin sponge particle slurry for tract embolization resulted in markedly reduced rates of pneumothorax and chest tube placement [18–21]. However, the number of patients with emphysema in these studies was small, and patients with emphysema were not classified; therefore, it is impossible to accurately assess the effect of tract embolization on the incidence of pneumothorax and chest tube placement in patients with different degrees of emphysema. Furthermore, given that the gelatin sponge particles were fabricated in-house in these studies, size consistency could not be ensured. Currently, there is no literature indicating that needle embolization with small gelatin sponge particles can cause ectopic embolization through tiny pulmonary veins. However, given our concerns regarding the potential for ectopic embolization, we employed homogeneous large gelatin sponge particles for needle embolization, and the results showed that no patients experienced ectopic embolization events. In the current study, we not only expanded the population of patients with emphysema but also stratified the patients according to the degree of emphysema, which is conducive to evaluating the application of tract embolization during PTLB in patients with emphysema. However, a few patients had severe emphysema (grades confluent CLE&ADE); therefore, we combined these into a single grade for analysis. There was no significant difference between the two groups in the confluent CLE&ADE grade of emphysema. However, fewer patients developed pneumothorax and required chest tube placement in the tract embolization group than in the non-tract embolization group.

Our results showed that the incidence of pneumothorax and chest tube placement increased with the length of the lung parenchyma that was punctured, regardless of whether gelatine sponge particles were used for tract embolization, consistent with the results of previous studies [19, 21]. Accordingly, the incidences of pneumothorax and chest tube placement increased with the severity of emphysema, regardless of whether tract embolization was performed. However, the incidence of pneumothorax and chest tube placement in the tract embolization group was reduced by approximately half when compared with that in the non-tract embolization group, and there was no statistical difference between the two groups in terms of complications other than pneumothorax. These results suggest that this method is safe and effective for reducing pneumothorax and chest tube placement.

This study has several limitations. First, this was a single-center retrospective study. Second, the severity of emphysema was determined based on a subjective assessment performed by a radiologist, which may have led to low reproducibility. In addition, owing to the advanced planning of the puncture path, crossing the pleura multiple times was only necessary for a few patients; therefore, we did not review the number of needle adjustments after passing through the pleura to reach the lesion. Moreover, the relatively small number of patients with severe emphysema may have limited our ability to assess PTLB-associated pneumothorax. Large-sample multicenter prospective studies are needed to verify these results.

## Conclusions

In conclusion, tract embolization using a gelatin sponge particle slurry after PTLB can be considered a safe and effective method to substantially reduce the incidence of postprocedural pneumothorax and chest tube placement in patients with emphysema.

## Data Availability

The datasets used and/or analysed during the current study available from the corresponding author on reasonable request.

## Abbreviations

ADE: advanced destructive emphysema
CLE: centrilobular emphysema
CT: computed tomography
PACS: Picture Archiving and Communication System
PTLB: percutaneous lung biopsy
TBB: Trans Bronchial Biopsy
TBNA: Trans Bronchial Needle Aspiration
